# Brain-derived neurotrophic factor contributes to neurogenesis after intracerebral hemorrhage: a rodent model and human study

**DOI:** 10.3389/fncel.2023.1170251

**Published:** 2023-05-11

**Authors:** Ting-Chun Lin, Yi-Chieh Tsai, Yun-An Chen, Tai-Horng Young, Chung-Che Wu, Yung-Hsiao Chiang, Chia-Hsin Kao, Abel Po-Hao Huang, Yi-Hua Hsu, Kai-Yun Chen, Li-Kai Tsai

**Affiliations:** ^1^Graduate Institute of Clinical Medicine, College of Medicine, National Taiwan University, Taipei, Taiwan; ^2^Department of Neurology and Stroke Center, National Taiwan University Hospital, Taipei, Taiwan; ^3^Institute of Biomedical Engineering, College of Medicine and College of Engineering, National Taiwan University, Taipei, Taiwan; ^4^Department of Surgery, School of Medicine, College of Medicine, Taipei Medical University, Taipei, Taiwan; ^5^Department of Neurosurgery, Taipei Medical University Hospital, Taipei, Taiwan; ^6^TMU Neuroscience Research Center, Taipei Medical University, Taipei, Taiwan; ^7^Taipei Neuroscience Institute, Taipei Medical University, Taipei, Taiwan; ^8^Department of Surgery, National Taiwan University Hospital, Taipei, Taiwan; ^9^Ph.D. Program in Medical Neuroscience, College of Medical Science and Technology, Taipei Medical University, Taipei, Taiwan; ^10^Department of Neurology, National Taiwan University Hospital Hsin-Chu Branch, Hsinchu, Taiwan

**Keywords:** cerebrospinal fluid, intracerebral hemorrhage (ICH), neurogenesis, subventricular zone (SVZ), brain-derived neurotrophic factor (BDNF)

## Abstract

**Background and purpose:**

Intracerebral hemorrhage (ICH) enhances neurogenesis in the subventricular zone (SVZ); however, the mechanism is not fully understood. We investigated the role of brain-derived neurotrophic factor (BDNF) in post-ICH neurogenesis in a rodent model and in patients with ICH using cerebrospinal fluid (CSF).

**Methods:**

A rat model of ICH was constructed via stereotaxic injection of collagenase into the left striatum. Patients with ICH receiving an external ventricular drain were prospectively enrolled. CSF was collected from rats and patients at different post-ICH times. Primary cultured rat neural stem cells (NSCs) were treated with CSF with or without BDNF-neutralized antibody. Immunohistochemistry and immunocytochemistry were used to detect NSC proliferation and differentiation. The BDNF concentration in CSF was quantified using enzyme-linked immunosorbent assays (ELISA).

**Results:**

In the rat model of ICH, the percentage of proliferating NSCs and neuroblasts in SVZ was elevated in bilateral hemispheres. The cultured rat NSCs treated with CSF from both rats and patients showed an increased capacity for proliferation and differentiation toward neuroblasts. BDNF concentration was higher in CSF collected from rats and patients with ICH than in controls. Blocking BDNF decreased the above-noted promotion of proliferation and differentiation of cultured NSCs by CSF treatment. In patients with ICH, the BDNF concentration in CSF and the neurogenesis-promoting capacity of post-ICH CSF correlated positively with ICH volume.

**Conclusion:**

BDNF in CSF contributes to post-ICH neurogenesis, including NSC proliferation and differentiation toward neuroblasts in a rat model and patients with ICH.

## Introduction

The adult mammalian brain comprises a population of neural stem cells (NSCs) in the subventricular zone (SVZ) adjacent to the lateral ventricles wall (Altman and Das, 1965; Morshead et al., 1994). After ischemic stroke, adult SVZ NSCs are activated to proliferate and differentiate toward doublecortin (DCX) -expressing neuroblasts, which are capable of migrating into the infarcted regions (Arvidsson et al., 2002; Parent et al., 2002; Yamashita et al., 2006). In a mouse model of ischemic stroke, conditional ablation of neuroblasts diminished post-stroke motor and cognitive functional improvement, implying that stroke-induced neurogenesis plays an important role in functional recovery (Sun et al., 2013). Intracerebral hemorrhage (ICH) also enhances neurogenesis and neuroblasts migration in hemorrhagic stroke; (Masuda et al., 2007; Otero et al., 2012) however, the mechanism underlying post-ICH neurogenesis is not fully understood and the features of neurogenesis in patients with ICH are unclear, possibly because non-invasive techniques to analyze neurogenesis are still limited.

Notably, the NSCs and ependymal cells at the SVZ form a pinwheel structure with an apical process in direct contact with cerebrospinal fluid (CSF), which regulates NSCs behavior through specific growth factors and signals (Mirzadeh et al., 2008; de Sonnaville et al., 2020). Our previous study using CSF from patients with another type of hemorrhagic stroke, subarachnoid hemorrhage (SAH), demonstrated that more proliferation-promoting factors in CSF were associated with better functional outcomes in SAH patients (Chen et al., 2018). Taken together, CSF is an attractive resource for the investigation of neurogenesis in both rodents and humans.

In addition, brain-derived neurotrophic factor (BDNF), a member of the neurotrophic factor family, participate in the survival, differentiation, and synaptic plasticity of neurons (Barde, 1989). BDNF supplement has been shown to promote functional recovery in a rat model of ICH (Chen et al., 2012; Ahn et al., 2021). Nevertheless, the role of endogenous BDNF in post-ICH neurogenesis in rodents and patients still requires further study. The present study aimed to investigate the contribution of BDNF in post-ICH neurogenesis using CSF collected from a rat model and patients with ICH.

## Materials and methods

### Animal model

Adult Sprague–Dawley (SD) rats aged 8 weeks (280–300 g) were subjected to induction of ICH. Using a microinjector pump, 0.2 U of bacterial collagenase (type VII-S, Sigma-Aldrich, St. Louis, MO, USA) in 1 μL saline was infused into the left striatum adjacent to the SVZ (1 mm anterior, 3.3 mm lateral, and 4.5 mm ventral relative to bregma) at a constant rate of 0.1 μL/min, leaving the needle in place for an additional 10 min after injection. In sham-operated controls, 1 μL saline was injected using the same procedures. Subsequently, the scalp was closed with sutures, and the rats were placed in a cage under an infrared heating lamp until recovery from anesthesia.

For collection of rat CSF samples, the cisterna magna was punctured using a U-100 insulin syringe with 28G × 1/2-inch needle (BD Biosciences, Franklin Lakes, NJ, USA) and 0.1–0.2 ml of CSF was then gently aspirated. CSF samples were centrifuged at 800 *g* RCF for 10 min at 4°C to avoid blood contamination and were then aliquoted and stored at −80°C. The animal experiment protocol was approved by the Institutional Animal Care and Use Committee at National Taiwan University (IACUC-20180009) and Taipei Medical University (LAC-2019-0236).

### Patients

A total of 15 patients who had been diagnosed with supra- or infratentorial ICH with intraventricular hemorrhage and had undergone external ventricular drain surgery between August 2017 and April 2020 were prospectively included. Among the 15 patients, 7 patients whose CSF samples could not be obtained at least 7 days after ICH were excluded and CSF samples from 8 patients were analyzed. The ICH volume was measured from initial computed tomography (CT) imaging. The functional outcome was evaluated at post-ICH 3 months using the modified Rankin Scale (mRS). CSF was also collected from eight patients with normal pressure hydrocephalus (NPH) to serve as controls. The human study was approved by the institutional review board of Taipei Medical University (TMU-JIRB N201703066) and the included patients all provided signed informed consent.

### Primary rat neural stem cell culture

Cortical NSCs were obtained from pregnant SD rats at the gestational age of 17 days according to our previously described protocol (Lee et al., 2016). Briefly, after dissecting rat embryos and transferring them into cold sterile HBSS (Gibco, Thermo Fisher Scientific, St. Louis, MO, USA), the embryonic cerebral cortices were separated from the brain, washed with PBS, triturated to create a single-cell suspension, and cultured in the complete media containing Dulbecco’s Modified Eagle Media (DMEM)/F-12 (Gibco) supplemented with 1% N2 supplement (Gibco), 20 ng/ml basic fibroblast growth factor (bFGF, Gibco), and 1% antibiotic-antimycotic solution (Gibco) at 37°C in a humidified atmosphere and 5% CO2 for 5 days, during which time primary neurospheres would be expected to form. For NSC differentiation analysis, neurospheres were subcultured on 12-well plates with coverslips pre-coated with poly-D-lysine (100 μg/mL, Sigma-Aldrich) and laminin (10 μg/mL, Sigma-Aldrich). The optimal condition of CSF treatment was pretested with different dosages of CSF to determine CSF saturation concentration in the above-noted neurosphere assay. Subsequently, cultured NSCs were treated with 1.0% rat CSF or 0.5% human CSF with or without BDNF antibody (3 μg/mL, AB1513P, Sigma-Aldrich) and IgG isotype control (3 μg/mL, catalog #31243, Invitrogen, Waltham, MA, USA) for 3 days and processed for immunocytochemistry.

### Immunohistochemistry

Rats at post-ICH days 1, 3, 5, and 7 were perfused transcardially with cold phosphate-buffered saline (PBS) followed by 10% phosphate buffered formalin. Brains were then removed and placed in 10% phosphate buffered formalin overnight at 4°C, 15% sucrose for 1 day, then 30% sucrose containing 0.1% sodium azide, and stored at 4°C until sectioning was processed. Tissue blocks were embedded in OCT Tissue-Tek (Sakura Finetek, St. Torrance, CA, USA) and cut into a series of 14 μm-thick coronal slices. Brain sections were permeabilized with 0.2% Triton X100 in PBS at room temperature for 15 min, fixed with 10% MeOH and 3% H_2_O_2_ in 0.2% Triton X100 in PBS for 15 min, and non-specific binding was blocked using blocking solution containing 3% normal donkey serum and 3% bovine serum albumin (BSA) in PBS for 1 h. Dual-staining was performed using anti-Ki67 (1:200, abcam, Cambridge, UK) and anti-nestin (1:1000, abcam) antibodies to label proliferating NSCs, and using anti-doublecortin (DCX) (1:500, abcam) and anti-glial fibrillary acidic protein (GFAP) (1:200, Sigma-Aldrich) antibodies to study NSC differentiation. After incubation with the above primary antibodies overnight at 4°C, samples were incubated with Alexa Fluor^®^ 594 and 488 (1:200, abcam) secondary antibodies. Nuclei were counterstained with DAPI (GeneTex, Irvine, CA, USA) and the slides were imaged using the fluorescence microscope (TissueFAXS, TissueGnostics, Vienna, Austria).

Immunoreactivity was quantified at the anterior dorsolateral region of SVZ, where the most active neurogenesis occurs at different post-ICH times (Falcão et al., 2012). The percentage of proliferating NSCs at SVZ was presented as the number of Ki67-positive and nestin-positive cells divided by the number of DAPI-positive cells. The ratio of DCX-positive cells divided by the number of DAPI-positive cells representing NSC differentiation toward neuroblasts, and the ratio of GFAP-positive area to perihematomal area representing NSC differentiating toward astrocytes or local astrocytosis, were also calculated. All values were then averaged to obtain final results for each condition.

### Immunocytochemistry

Cultured NSCs were fixed with ice-cold methanol for 20 min, permeabilized with PBS containing 0.25% Triton X-100 (PBST) for 10 min, blocked with 1% BSA in PBST for 30 min at room temperature, and double stained with primary antibodies (Ki67 1:500 and nestin 1:200; GFAP 1:300 and DCX 1:200) overnight at 4°C. After washing, cells were incubated with secondary antibodies (1:200, abcam) and counterstained with DAPI (GeneTex). Images were captured using the fluorescence microscope (Zeiss Axio Observer 7 or Zeiss AxioVert 200M, Zeiss). For each condition, Ki67-positive, nestin-positive and DCX-positive cells were counted and divided by the DAPI-positive cells and calculated at least three neurospheres per coverslip. GFAP expression was determined by a GFAP-positive area divided from the whole area of at least three neurospheres per coverslip. All values were then averaged to obtain a final immunoactivity level for each condition.

### ELISA analysis of BDNF

Enzyme-linked immunosorbent assay (ELISA) kits (DBNT00, R&D Systems, Minneapolis, MN, USA) were used to detect total BDNF protein concentration in CSF per manufacturer’s instructions. All samples were tested in duplicate, and the optical density was read at 450 nm on a microplate reader (Multiskan GO, Thermo Scientific, Waltham, MA, USA).

### Statistical analysis

Data are presented as mean ± standard error of the mean (SEM). Statistical analysis comparing mean differences was performed using analysis of variance (ANOVA) followed by *post-hoc* analysis using Tukey’s test. Correlations between ICH volume and BDNF concentration in CSF and the neurogenesis-promoting capacity of post-ICH CSF were examined using simple linear regression analysis. All statistical analyses were performed using the GraphPad Prism software (GraphPad Software, Inc., San Diego, CA, USA). All tests of significance were 2-tailed with a threshold for significance of *p* < 0.05.

## Results

### ICH promotes neurogenesis in bilateral SVZ of rats

**FIGURE 1:**
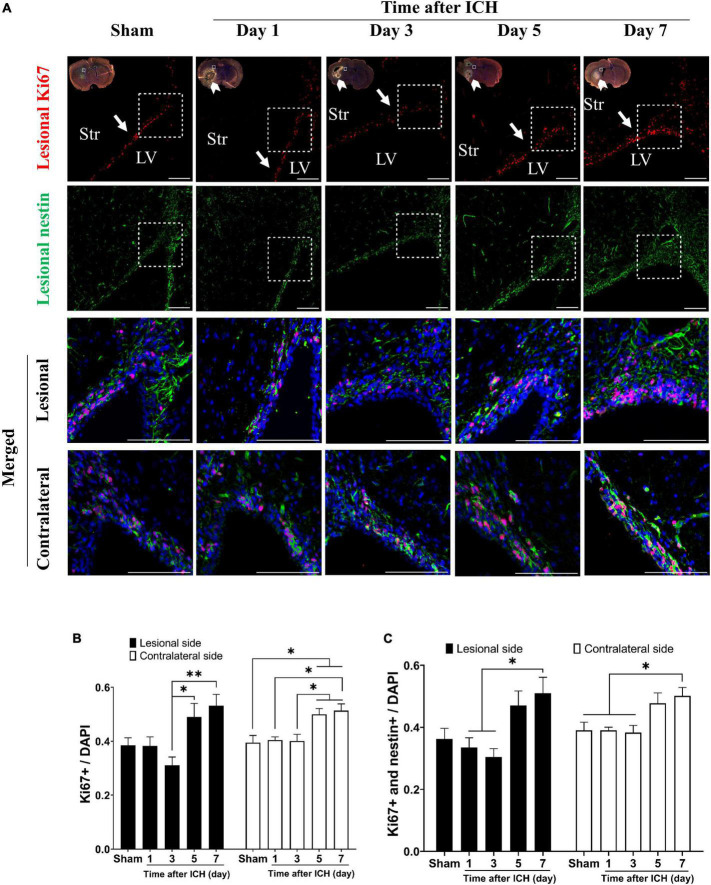
Proliferation of neural stem cells (NSCs) in subventricular zone (SVZ) of rats with intracerebral hemorrhage (ICH). **(A)** Immunofluorescent double labeling for Ki67 (red) and nestin (green) on cryostat coronal sections of rat brain around SVZ at different post-ICH times was shown. Nuclei were counterstained with DAPI (blue). White rectangles indicate the region around upper lateral SVZ in the lesional hemisphere. White arrowheads indicate location of ICH and white arrows indicate SVZ. The lesional merged image depicts the magnified area shown in the white dotted rectangle. Scale bars = 100 μm. **(B)** The proliferating cells in the SVZ were detected with Ki67 and DAPI double staining. The percentage of proliferating cells among all cells in SVZ was increased on post-ICH days 5 and 7 in bilateral hemispheres. **(C)** The proliferating NSCs were detected with Ki67, nestin, and DAPI triple staining. The percentage of proliferating NSCs among all cells in SVZ was increased on post-ICH day 7 in bilateral hemispheres. Data are represented as mean ± standard error of mean (SEM), *n* = 4 for each time-point. *P < 0.05; ^**^P < 0.01, one-way ANOVA with Tukey’s multiple comparisons test. LV, lateral ventricle; Str, striatum.

The results of brain sections in SVZ from rats at post-ICH days 1, 3, 5, and 7 were analyzed and compared to those from sham controls. The proliferating cells in the SVZ were detected with Ki67 and DAPI double staining. Compared to all cells in the SVZ, the percentage of proliferating cells in the SVZ lesional side was relatively increased on post-ICH days 5 and 7 (Figures 1A, B). While the proliferating NSCs were detected with Ki67, nestin and DAPI triple staining, the percentage of proliferating NSCs among all cells was relatively increased on post-ICH day 7 in the lesional side of the SVZ (Figures 1A, C). Notably, similar increases in the percentage of proliferating cells or proliferating NSCs in SVZ on post-ICH days 5 and 7 were also detected on the contralateral side.

**FIGURE 2:**
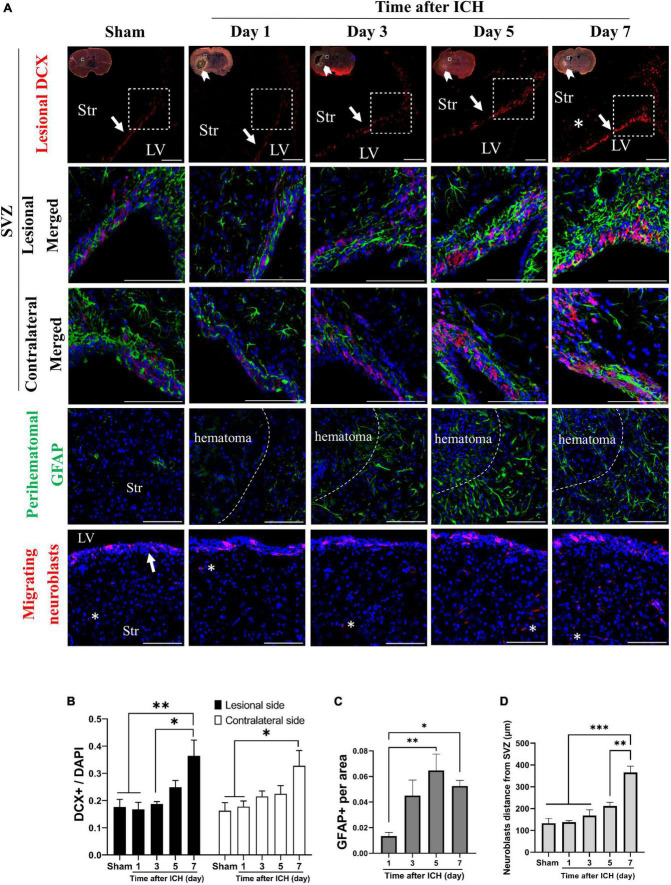
Differentiation of neural stem cells (NSCs) in rats with intracerebral hemorrhage (ICH). **(A)** Immunofluorescent labeling for doublecortin (DCX, red) and GFAP (green) on cryostat coronal sections of rat brain at different post-ICH times was shown. Nuclei were counterstained with DAPI (blue). White rectangles indicate the region around upper lateral SVZ in the lesional hemisphere. White arrowheads indicate location of ICH and white arrows indicate SVZ. The lesional merged image depicts the magnified area shown in the white dotted rectangle. **(B)** The neuroblasts were detected with DCX staining. The number of neuroblasts was increased on post-ICH day 7 in bilateral hemispheres. **(C)** The number of GFAP immunoreactive cells was increased on post-ICH days 5 and 7 in the perihematomal region. **(D)** Neuroblasts (asterisk) migrated longer distance on post-ICH day 7 than other post-ICH times in the lesional striatum. Data are represented as mean ± SEM, *n* = 4 for each time-point in panels **(B,C)** and *n* = 3 for each time-point in panel **(D)**. *P < 0.05; ^**^P < 0.01, ^***^P < 0.001, one-way ANOVA with Tukey’s multiple comparisons test. LV, lateral ventricle; Str, striatum. Scale bars = 100 μm.

In addition, the cell density of neuroblasts (DCX immunoreactive cells) in SVZ and astrocytes (GFAP immunoreactive cells) in the perihematomal region were analyzed. In the lesional side, the number of neuroblasts at SVZ and astrocytes in perihematomal region were both increased on post-ICH day 7 and day 5, respectively (Figures 2A–C). The farthest distance neuroblasts migrated from the SVZ toward the striatum was also measured. On post-ICH day 7, neuroblasts showed a longer migratory distance than those at other post-ICH times on the lesional side (Figure 2D). Similar increases in the number of neuroblasts were detected at the contralateral SVZ, but an increase in the number of astrocytes and the migratory distance of neuroblasts was not found in the contralateral striatum. Since CSF communicates in bilateral lateral ventricles and contacts bilateral SVZs directly, the simultaneous activation of NSC proliferation and differentiation toward neuroblasts in bilateral SVZs upon unilateral ICH implies that certain factors in the CSF may contribute to the above-noted neurogenesis.

### Post-ICH CSF promotes proliferation and differentiation of cultured NSCs

**FIGURE 3:**
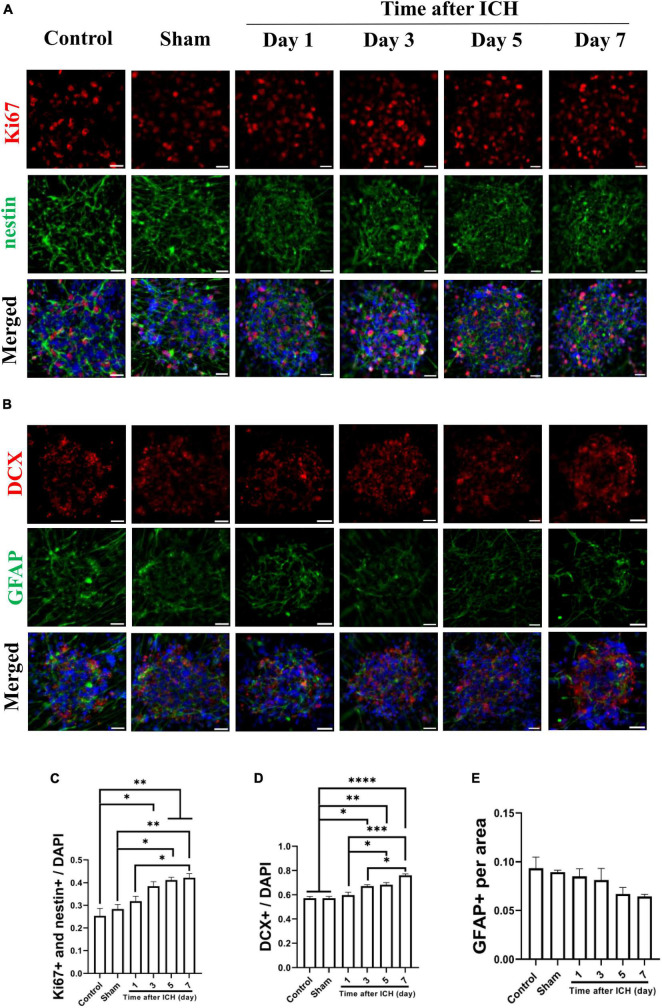
Cerebrospinal fluid (CSF) collected from post-ICH rats promotes proliferation and differentiation of cultured neural stem cells (NSCs). **(A)** Cultured NSCs were treated with CSF collected from post-ICH rats at different times, followed by immunostaining labeling of Ki67 (red), nestin (green) and DAPI (blue) for analysis of NSC proliferation. **(B)** The differentiation of cultured NSCs was determined by immunofluorescent labeling for DCX (red), GFAP (green), and DAPI (blue). **(C)** The percentage of double Ki67 and nestin immunoreactive NSCs among all cells was increased after treatment with CSF collected from rats on post-ICH days 3 to 7. **(D)** The percentage of DCX immunoreactive cells in neurospheres was increased after treatment with CSF collected from rats on post-ICH days 3 to 7. **(E)** The percentage of GFAP immunoreactive cells in neurospheres did not change after CSF treatment. Data are represented as mean ± SEM, *n* = 3 for each condition. *P < 0.05; **P < 0.01, ***P < 0.001, ****P < 0.0001. One-way ANOVA with Tukey’s multiple comparisons test. Scale bar = 20 μm.

Cerebrospinal fluid was collected from a rat model of ICH at different post-ICH times. The primary cultured rat embryonic cortical NSCs were then treated with CSF to test the hypothesis that post-ICH CSF promotes neurogenesis *in vitro*. Under treatment with CSF collected from rats at post-ICH days 3, 5, and 7, Ki67 and nestin double immunoreactive cells increased in neurospheres compared with those in the control group, indicating that post-ICH CSF can stimulate proliferation of cultured NSCs (Figures 3A, C). In addition, the number of neuroblasts, as determined by DCX immunostaining, was also increased after treatment with CSF collected from rats at post-ICH days 3, 5, and 7 (Figures 3B, D). However, no significant changes were noted in the character of astrocytes as determined by GFAP immunostaining after CSF treatment (Figures 3B, E).

**FIGURE 4:**
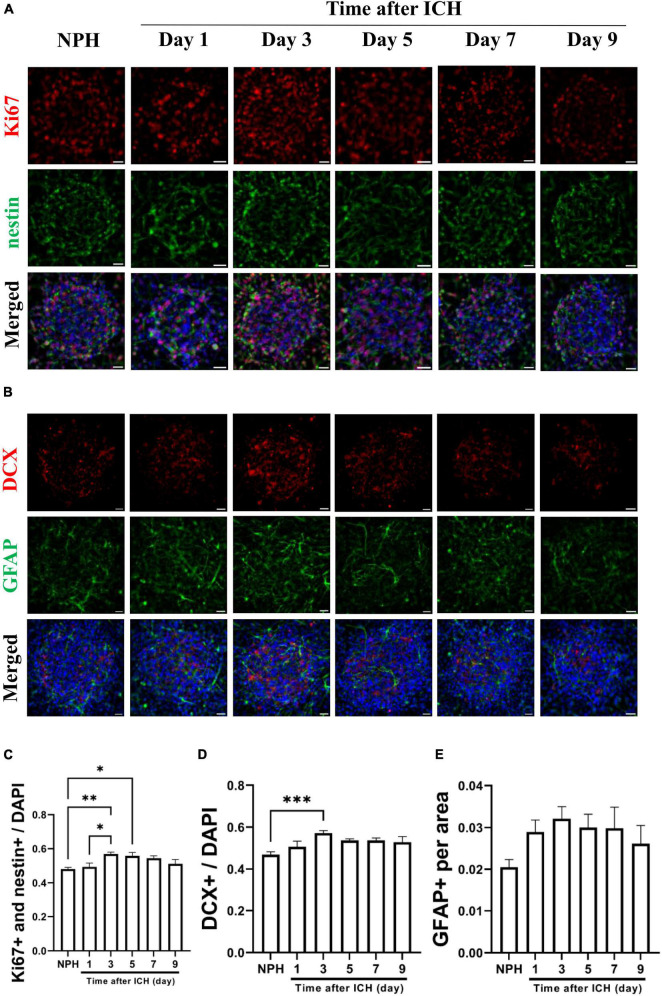
Cerebrospinal fluid (CSF) collected from patients with intracerebral hemorrhage (ICH) promotes proliferation and differentiation of cultured NSCs. **(A)** Cultured NSCs were treated with CSF collected from patients with ICH at different post-ICH times, followed by immunostaining labeling of Ki67 (red), nestin (green) and DAPI (blue) for analysis of NSC proliferation. Treatment with CSF collected from patients with normal pressure hydrocephalus (NPH) was defined as control. **(B)** The differentiation of cultured NSCs was determined by immunofluorescent labeling for DCX (red), GFAP (green), and DAPI (blue). **(C)** The percentage of double Ki67 and nestin immunoreactive NSCs among all cells was increased after treatment with CSF collected from patients on post-ICH days 3 and 5. **(D)** The percentage of DCX immunoreactive cells in neurospheres was increased after treatment with CSF collected from patients on post-ICH day 3. **(E)** The percentage of GFAP immunoreactive cells in neurospheres did not change after CSF treatment. Data are represented as mean ± SEM, *n* = 8 for NPH and days 3 to 7, *n* = 7 for day 1 and *n* = 6 for day 9. *P < 0.05; ^**^P < 0.01, ^***^P < 0.001. One-way ANOVA with Tukey’s multiple comparisons test. Scale bar = 20 μm.

Additional CSF samples were collected from patients with ICH at different post-ICH times to treat cultured NSCs for studying the effects of human post-ICH CSF on neurogenesis. Treatment with CSF collected from patients with NPH was defined as the control group. Under treatment with CSF collected from patients at post-ICH days 3 and 5, Ki67 and nestin double immunoreactive cells increased in neurospheres compared with those in the control group, implying that post-ICH human CSF enhances proliferation of cultured NSCs (Figures 4A, C). In addition, the number of neuroblasts was also increased after treatment with CSF collected from patients at post-ICH day 3 (Figures 4B, D). No significant changes were noted in the character of astrocytes after CSF treatment (Figures 4B, E). Taken together, CSF collected from both post-ICH rats and patients induces NSCs to proliferate and differentiate toward neuroblasts in cultured neurospheres.

### BDNF in post-ICH CSF contributes to neurogenesis of culture NSCs

**FIGURE 5:**
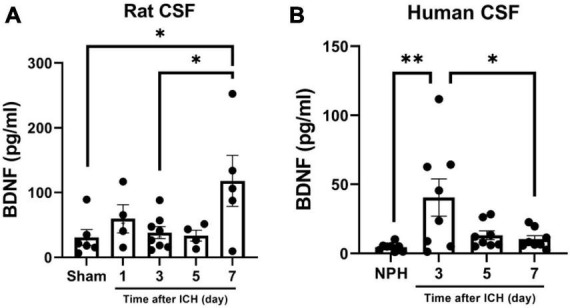
Brain-derived neurotrophic factor (BDNF) concentration in cerebrospinal fluid (CSF) collected from rats and patients with intracerebral hemorrhage (ICH). **(A)** The concentration of BDNF analyzed using ELISA was increased in CSF collected from rats on post-ICH day 7. **(B)** The concentration of BDNF was increased in CSF collected from patients on post-ICH day 3. Concentration of BDNF in CSF collected from patients with NPH was defined as control. Data are represented as mean ± SEM. *P < 0.05; ^**^P < 0.01, one-way ANOVA with Tukey’s multiple comparisons test.

Since BDNF is a major enhancer of neurogenesis upon cerebral ischemic insults, and BDNF in CSF contributes to neurogenesis in SAH (Chen et al., 2013; Lee et al., 2016), the concentration of BDNF in CSF collected from rats and patients at different post-ICH times were analyzed using ELISA. The concentration of BDNF in the CSF of rats collected on post-ICH day 7 was higher than that from sham control rats (118.0 ± 39.5 vs. 30.9 ± 12.2 pg/ml; *P* = 0.036) (Figure 5A). Besides that result, the concentration of BDNF in CSF of patients collected on post-ICH day 3 was also higher than that from patients with NPH (40.4 ± 13.5 vs. 4.6 ± 1.0 pg/ml; *P* = 0.007) (Figure 5B).

**FIGURE 6:**
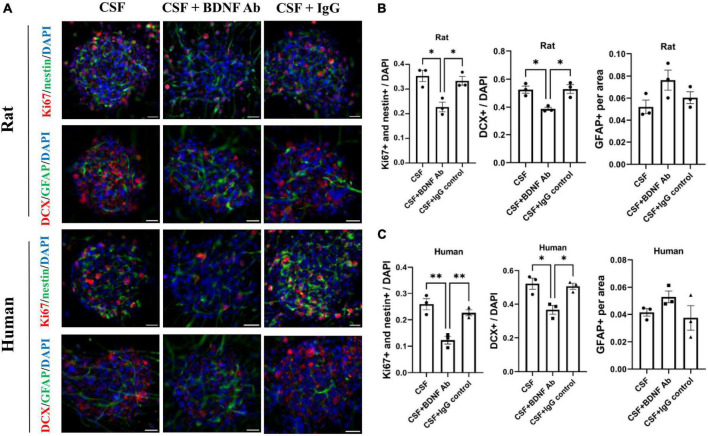
Blocking Brain-derived neurotrophic factor (BDNF) decreases promotion in proliferation and differentiation of cultured NSCs by CSF treatment. **(A)** Cultured NSCs were treated with CSF collected from rats or patients with ICH with or without BDNF neutralized antibody and IgG control antibody. The proliferation of NSCs was determined by immunofluorescent labeling for Ki67 (red) and nestin (green), while the differentiation was determined by immunofluorescent labeling for DCX (red) and GFAP (green) with DAPI (blue). **(B,C)** Blocking BDNF decreased proliferation and differentiation toward neuroblasts of NSCs under treatment with CSF collected from rats or patients with ICH. Data are represented as mean ± SEM, *n* = 3 for each experiment. *P < 0.05; ^**^P < 0.01. One-way ANOVA with Tukey’s multiple comparisons test. Scale bar = 20 μm.

To confirm the contribution of BDNF on neurogenesis, the promoting capacity of post-ICH CSF on proliferation and differentiation of NSCs was examined to determine whether it would be eliminated by BDNF neutralizing antibody. IgG isotype antibody was used as the control. Under treatment with post-ICH CSF of rats, BDNF neutralizing antibody reduced the percentage of proliferating NSCs and neuroblasts in neurospheres, while IgG isotype antibody showed no similar reduction of treatment effects (Figures 6A, B). In addition, under treatment with post-ICH CSF of patients, BDNF neutralizing antibody also decreased the percentage of proliferating NSCs and neuroblasts, and a similar decrease was not detected in treatment with IgG isotype antibody (Figures 6A, C). On the other hand, BDNF neutralizing antibody did not change the percentage of astrocytes significantly upon treatment with post-ICH CSF from rats or patients. Taken together, these results suggest that BDNF induced after ICH plays a role in post-ICH NSC proliferation and differentiation toward neuroblasts in both rats and patients.

### ICH volume is associated with BDNF concentration and neurogenesis capacity

Eight patients with ICH (aged 62.4 ± 10.7 years; men 62.5%) who were required to receive an external ventricular drain were enrolled (Table 1). The ICH was located at the cerebellum (*n* = 5), thalamus (*n* = 2) and putamen (*n* = 1), with mean hematoma volume 10.1 ± 7.8 ml. Most patients (87.5%) showed unfavorable outcomes at post-ICH 3 months (mRS > 2). The mean BDNF concentration in CSF, collected from patients at post-ICH day 3, was 41.3 ± 14.8 pg/ml.

**TABLE 1:** Clinical characteristics of patients with intracerebral hemorrhage.

Patient no.	Age (year)	Sex	ICH location	ICH volume (ml)	BDNF (pg/ml)	3-month mRS
1	43	F	Cerebellum	7.9	5.2	3
2	73	M	Putamen	28.0	125.0	4
3	64	M	Cerebellum	5.9	39.5	2
4	78	M	Thalamus	5.1	62.7	5
5	61	F	Thalamus	3.1	8.9	5
6	58	F	Cerebellum	13.5	1.3	5
7	70	M	Cerebellum	3.7	23.5	5
8	52	M	Cerebellum	13.9	64.3	3

BDNF concentration was analyzed in CSF collected from patients at post-ICH day 3. BDNF, brain-derived neurotrophic factor; F, female; ICH, intracerebral hemorrhage; M, male; mRS, modified Rankin Scale.

**FIGURE 7:**
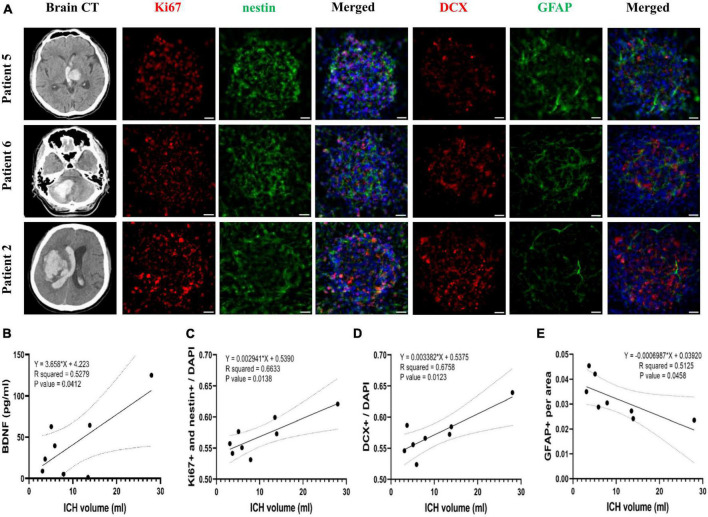
The correlation of Brain-derived neurotrophic factor (BDNF) concentration and neurogenesis promoting capacity of post-ICH CSF with intracerebral hemorrhage (ICH) volume in patients. **(A)** Cultured neural stem cells (NSCs) were treated with CSF collected from patients with ICH for 3 days. The proliferation of NSCs was determined by immunofluorescent labeling for Ki67 (red) and nestin (green), while the differentiation was determined by immunofluorescent labeling for DCX (red) and GFAP (green) with DAPI (blue). The ICH volume was measured using initial head CT scan. **(B)** The BDNF concentration in CSF was positively correlated with ICH volume. **(C)** The percentage of proliferating NSCs among all cells was positively correlated to ICH volume. **(D)** The percentage of neuroblasts was positively correlated to ICH volume. **(E)** The percentage of astrocytes was negatively correlated to ICH volume. Scale bar = 20 μm.

Attempts to correlate the ICH volume with BDNF concentration and neurogenesis promoting capacity of CSF revealed that the initial ICH volume correlated positively with BDNF concentration in CSF collected from patients at post-ICH day 3 (*R*^2^ = 0.53, *P* = 0.041) (Figure 7B). After further treatment of the cultured rat NSCs with CSF collected from patients at post-ICH day 3, the ICH volume also correlated positively with the capacity of NSC proliferation (*R*^2^ = 0.66, *P* = 0.014) (Figures 7A, C) and differentiation toward neuroblasts (*R*^2^ = 0.68, *P* = 0.012) (Figures 7A, D), but was negatively associated with the capacity of NSC differentiation toward astrocytes (*R*^2^ = 0.52, *P* = 0.046) (Figures 7A, E). Therefore, the CSF collected from patients with larger ICH volume has higher concentration of BDNF and shows a higher capacity to promote NSC proliferation and differentiation toward neuroblasts rather than astrocytes. However, the ICH volume (*R*^2^ = 0.01, *P* = 0.78), BDNF concentration in CSF (*R*^2^ = 0.02, *P* = 0.73), and neurogenesis promoting capacity of CSF (proliferation: *R*^2^ = 0.08, *P* = 0.51; differentiation toward neuroblast: *R*^2^ = 0.05, *P* = 0.60) did not correlate with functional outcomes evaluated by mRS at post-ICH 3 months.

## Discussion

Although post-ICH neurogenesis is well known from studies using rodent models (Masuda et al., 2007; Otero et al., 2012), the mechanism behind post-ICH neurogenesis is still unclear and the feature of neurogenesis in patients with ICH is limited. The present study demonstrated that ICH enhanced NSC proliferation and differentiation toward neuroblasts not only in the lesional SVZ, but also in the contralateral SVZ of a rat model of ICH. CSF collected from either post-ICH rats or patients were shown to induce cultured NSCs to proliferate and differentiate toward neuroblasts. BDNF concentration increased in CSF collected from both rats and patients with ICH. BDNF neutralizing antibody eliminated the promoting capacity of post-ICH CSF from rats or patients on cultured NSC proliferation and differentiation to the neural lineage. In addition, CSF collected from patients with larger ICH volume had higher concentration of BDNF and showed a higher capacity to promote NSC proliferation and to differentiate toward neuroblasts rather than astrocytes. These results indicate that BDNF in CSF contributes to post-ICH neurogenesis in a rat model and in patients with ICH.

In line with prior studies (Masuda et al., 2007; Otero et al., 2012), the present study found that ICH stimulated neurogenesis in bilateral SVZs. NSCs at SVZ extend a thin cellular apical process between ependymal cells reaching the ventricular lumen and contact with CSF, which provides extrinsic regulatory cues to the SVZ niche (Sanai et al., 2004; Mirzadeh et al., 2008). Since CSF communicates in bilateral lateral ventricles and contacts SVZ directly, factors in CSF are likely contribute to neurogenesis in bilateral SVZs upon unilateral ICH. Treatment with CSF collected from both post-ICH rats and patients was further confirmed to enhance neurogenesis capacity of cultured rat NSCs to proliferate and differentiate toward neuroblasts. These results support the existence of certain neurogenesis-promoting factors in post-ICH CSF in a rodent model and in patients. Therefore, CSF is a valuable sample for investigation of cerebral neurogenesis and post-stroke stem cell biology.

Brain-derived neurotrophic factor exhibits strong neurotrophic factor signals, which induces neurogenesis in adult SVZ via tyrosine kinase B receptors or p75 neurotrophin receptors (Yan et al., 1994; Zigova et al., 1998; Young et al., 2007). Cultured NSCs treated with BDNF showed prolonged survival and preference of neuronal differentiation with a decrease in glial phenotype (Kirschenbaum and Goldman, 1995; Islam et al., 2009). Since our previous study found that SAH promotes proliferation, differentiation, and migration of NSCs via BDNF upregulation (Lee et al., 2016), this study focused on the role of BDNF in neurogenesis using post-ICH CSF. Notably, while the concentration of BDNF increased and reached peak in CSF from rats at post-ICH day 7, NSC proliferation and differentiation at SVZs in ICH rats were activated mainly at post-ICH day 7 and CSF collected from rats at post-ICH day 7 showed the highest capacity to promote proliferation and differentiation toward neuroblasts in cultured NSCs. In patients with ICH, the concentration of BDNF was at peak in CSF from patients at post-ICH day 3 and CSF collected from patients exclusively at post-ICH day 3 enhanced capacity of proliferation and differentiation toward neural lineage in cultured NSCs. In addition, BDNF neutralizing antibody eliminated the above promoting capacity of post-ICH CSF in neurogenesis. Therefore, BDNF was shown to play an important role in post-ICH neurogenesis. Previous studies also have shown that BDNF supplemented directly or through cell therapy promotes functional recovery in rodent models of ischemic and hemorrhagic stroke (Chen et al., 2012, 2013; Ahn et al., 2021). Therefore, BDNF-based therapy is a future potential treatment strategy for ICH via activation of neurogenesis.

Prior studies emphasizing human post-stroke neurogenesis remain rare, possibly because non-invasive techniques to analyze neurogenesis are still limited. The present study used post-ICH CSF from patients to indirectly investigate the features of post-ICH neurogenesis. We demonstrated that CSF samples collected from patients with larger ICH volume have higher BDNF concentration and higher capacity to promote NSC proliferation and differentiate toward neuroblasts rather than astrocytes. For the first time, the association between ICH volume and growth factor concentration as well as neurogenesis capacity in the brain was shown indirectly. Upon acute stroke, neurons and non-neuronal cells such as astrocytes, microglia, ependymal cells, and endothelial cells produce a substantial amount of BDNF (Béjot et al., 2011b). Notably, in ischemic stroke, the severity of cerebral infarct was not associated with BDNF expression either in rodent models or in patients (Béjot et al., 2011a; Karantali et al., 2021). However, in the present study, the ICH volume seems to correlate positively with BDNF concentration in CSF, implying that different pathophysiology is evident between ischemic and hemorrhagic stroke. Post-stroke outcomes have previously shown that SAH severity was associated with the proliferation capacity of cultured NSCs promoted by CSF collected from SAH patients, which correlated well with clinical outcomes (Chen et al., 2018). Although the present study also found a positive correlation between ICH volume and neurogenesis capacity of NSCs promoted by CSF collected from ICH patients, neither the ICH volume, BDNF concentration, or neurogenesis capacity correlated with functional outcomes. This may be attributed to the small sample size in the present study, the variable ICH location, and the universally unfavorable outcomes in most of the included patients. Further large studies are mandatory in order to revisit the relationship between neurogenesis capacity and stroke outcomes in patients with ICH.

This study has several limitations. First, because of ethics issues, we used CSF collected from patients with NPH as a control group rather than healthy subjects. The possibility of any differences in components between CSF from NPH patients and healthy controls cannot be excluded. Second, in order to collect serial CSF samples from external ventricular drain, enrolled patients with ICH should have received surgical intervention based on clinical requirements, for which we cannot rule out selection bias. Third, in patients with concomitant intraventricular hemorrhage, the experimental results collected from CSF samples may be influenced by blood contamination. However, cultured NSCs were treated with human red blood cells and plasma and no increases were found in the neurogenesis capacity of NSCs in treatment with lytic or non-lytic red blood cells or plasma, as previously described (Chen et al., 2018). Fourth, post-ICH neurogenesis in the brain was not directly analyzed; instead, the promoting capacity of CSF on NSC proliferation and differentiation was used to indirectly reflect the neurogenesis capacity of each individual. Since *in vivo* detection of cerebral neurogenesis is still highly limited, the method provided in the present study may remain an alternative for future investigation of post-stroke neurogenesis. Fifth, the mean concentration of BDNF in the CSF of rats (post-ICH day 7) was 2.86 times higher than in patients’ CSF (post-ICH day 3) (Figure 5). However, since the concentration for each sample was highly variable, the comparison of BDNF promoting capacity between rats and human upon ICH requires future studies to address. In addition, a type-II error may exist in the analysis of the reduction of GFAP area, which showed a clear trend in cultured rat NSCs treated with CSF from the rats (Figure 3E). Finally the number of patients was too few to perform comprehensive subgroup analysis or data correlation. Results from further large prospective studies are anticipated.

## Conclusion

Cerebrospinal fluid samples from a rat model and patients with ICH demonstrated that acute ICH induced BDNF production, which contributes to post-ICH neurogenesis in both NSC proliferation and differentiation toward neuroblasts. Results provide evidence for future BDNF-based therapy in patients with ICH via effects of augmenting endogenous neurogenesis.

## Data availability statement

The original contributions presented in this study are included in the article/supplementary material, further inquiries can be directed to the corresponding authors.

## Ethics statement

The studies involving human participants were reviewed and approved by the Institutional Review Board of Taipei Medical University (TMU-JIRB N201703066). The patients/participants provided their written informed consent to participate in this study. The animal study was reviewed and approved by the Institutional Animal Care and Use Committee at National Taiwan University (IACUC-20180009) and Taipei Medical University (LAC-2019-0236).

## Author contributions

T-CL and L-KT contributed to the conception and design of the study. T-CL, Y-AC, T-HY, AH, Y-HH, and L-KT organized the database. T-CL, Y-CT, C-CW, Y-HC, K-YC, and L-KT performed the statistical analysis. T-CL wrote the first draft of the manuscript. L-KT wrote sections of the manuscript. All authors contributed to manuscript revision, read, and approved the submitted version.
